# Early Eye Disengagement Is Regulated by Task Complexity and Task Repetition in Visual Tracking Task

**DOI:** 10.3390/s24102984

**Published:** 2024-05-08

**Authors:** Yun Wu, Zhongshi Zhang, Farzad Aghazadeh, Bin Zheng

**Affiliations:** 1 Department of Surgery, Faculty of Medicine and Dentistry, University of Alberta, 162A Heritage Medical Research Centre, 11207-87 Ave NW, Edmonton, AB T6G 2S2, Canada; yun.wu@ualberta.ca (Y.W.); zszhang@ualberta.ca (Z.Z.); 2 Department of Mechanical Engineering, University of Alberta, Edmonton, AB T6G2E1, Canada; farzad@ualberta.ca

**Keywords:** eye tracking, bio-signal interpretation, cognitive load, human-computer interaction, human factors

## Abstract

Understanding human actions often requires in-depth detection and interpretation of bio-signals. Early eye disengagement from the target (EEDT) represents a significant eye behavior that involves the proactive disengagement of the gazes from the target to gather information on the anticipated pathway, thereby enabling rapid reactions to the environment. It remains unknown how task difficulty and task repetition affect EEDT. We aim to provide direct evidence of how these factors influence EEDT. We developed a visual tracking task in which participants viewed arrow movement videos while their eye movements were tracked. The task complexity was increased by increasing movement steps. Every movement pattern was performed twice to assess the effect of repetition on eye movement. Participants were required to recall the movement patterns for recall accuracy evaluation and complete cognitive load assessment. EEDT was quantified by the fixation duration and frequency within the areas of eye before arrow. When task difficulty increased, we found the recall accuracy score decreased, the cognitive load increased, and EEDT decreased significantly. The EEDT was higher in the second trial, but significance only existed in tasks with lower complexity. EEDT was positively correlated with recall accuracy and negatively correlated with cognitive load. Performing EEDT was reduced by task complexity and increased by task repetition. EEDT may be a promising sensory measure for assessing task performance and cognitive load and can be used for the future development of eye-tracking-based sensors.

## 1. Introduction

The recognition and interpretation of human bio-signals represents a pivotal aspect in the development of wearable sensors. The use of eye-tracking technology can indicate a considerable amount of behavior-related information. When observing a moving object, human operators’ eyes are not merely fixed on the target; they intermittently disengage from the target and move ahead to gather information on the projected pathway [1]. The premature removal of the eye from a target is what we refer to in this paper as early eye disengagement from target (EEDT). EEDT also has some other synonyms in literature, such as look-ahead fixation or proactive eye movement. Subjects use this gaze in a task to search for specific information that is critical for planning and coordinating actions before they actively engage. This eye strategy is common and useful in activities such as driving, sports, and simple household tasks, and it minimizes the need for continuous use of working memory. It also increases the efficiency of actions by reducing temporal and spatial uncertainty, allowing more precise coordination of eye and hand movements [1,2,3,4].

EEDT can be measured through eye-tracking technology. For example, we consider a gaze shift from the tracked target to a region ahead of it as a performance of EEDT. The frequency and duration of these shifts provide quantitative measures of EEDT. Specific metrics might include the duration and quantity that the gaze spends ahead of the target. Additionally, the position of the gaze relative to the target and the timing of the initial gaze shift away from the target in relation to movement onset can be considered alternative options. EEDT is not incidental; it is driven by human cognition that allows a human operator to anticipate and estimate travel distance and change in movement direction [1,2,3,4]. EEDT serves as the foundation for a fast response to the environment and precise motor control. In some extremely high-risk and cognitively demanding conditions, performing EEDT leads to life-saving consequences [3,5,6,7].

Among a few papers reporting eye disengagement in performing a continuous goal-directed movement, Zheng reported that people performed eye disengagement from their current target earlier and with a longer duration if the task was relatively easy than if the task was more demanding [8]. However, direct evidence on how the task complexity of individuals modulates the EEDT has yet to be fully investigated [1]. In studying the impacts of human memory on eye scanning, Hannula et al. reported that an individual’s gaze pattern toward each stimulus unconsciously changes in response to pre-existing experience or memory [9]. Prior experience potentially alters the nature of perceptual processing, and these eye behaviors may be linked with the activation of the hippocampus in the brain [10,11]. Nonetheless, in studies regarding the relationship between eye scanning and memory, much of the available evidence is based on watching a static picture without involving any moving objects [9].

In surgical eye–hand coordination research, Zheng et al. revealed that experienced surgeons disengaged their eyes from the preceding subtask earlier, whereas novices disengaged their eyes with a significant delay [8]. Chainey et al. discovered that the eyes of skilled microsurgeons moved to the target board earlier than the tools in their hands, compared to microsurgery residents [12]. This evidence suggested that repeating the task may alter the way that eye disengagement from the current target is performed. However, the extent to which task repetition affects EEDT with moving targets has not been intentionally studied.

In this study, we asked people to watch a moving target on the monitor and examine the frequency and duration of EEDT performed under different experimental conditions. We aimed to investigate whether the performance of EEDT would be regulated by the change in task complexity. We also aimed to investigate how EEDT would be altered by repetition of the same task. In addition, we aimed to examine the interaction effect between task complexity and task repetition. The complexity of the arrow tracking task was regulated by varying the arrow moving lengths and direction changes. To investigate repetition-based effects on the EEDT, we asked human operators to perform the task twice within each task complexity. We hypothesized that as task complexity increased, the EEDT would decrease, measured by decreasing the number and duration of fixations located in the area before the moving target. We also hypothesized that task repetition would increase the EEDT, measured by increasing the number and duration of eye fixation in the area before the moving target. In addition, we hypothesized that the effect of repetition on performing EEDT would be constrained by the increasing task complexity.

## 2. Methods

This research was conducted at the Surgical Simulation Research Lab of the University of Alberta’s Department of Surgery. Ethics approval was obtained from the Health Research Ethics Board of the University of Alberta before the recruitment of participants. Our participants were healthy adults with normal vision or corrected to normal vision. Prior to the experiment, each participant provided informed written consent. This study utilized the same experimental setting as our prior work [13], with both projects stemming from the same research initiative and using the same group of participants’ eye-tracking datasets. While the previous publication focused on aspects of pupil variation, the current analysis focuses on a different aspect of EEDT, a gaze-based eye movement.

### 2.1. Apparatus

**Figure 1:**
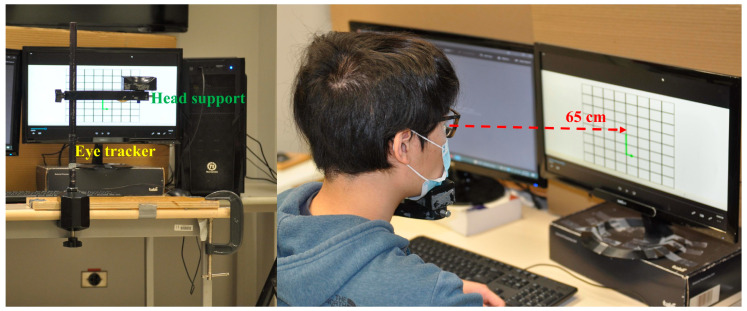
Apparatus setup. Blue text indicates the Tobii Pro Nano eye tracker; green text indicates the head support. Participants sat in front of the computer with their chin on the support scaffold. The red dashed arrows depict the approximate distance between the eye and the screen.

The visual stimuli in this experiment were delivered through a commercially available PC (Intel, Santa Clara, CA, USA) connected to a monitor with a size of 476 mm × 370 mm (ASUS Computer Inc., Taipei, Taiwan). Participants’ eye movement data were collected using a Tobii Pro Nano (Tobii Technology Inc., Stockholm, Sweden), a remote and screen-based eye tracker with a sampling rate of 60 Hz and a reported accuracy of 0.5 degrees. Data were analyzed using the Tobii Pro Lab 2021 software. The accompanying monitor displayed a resolution of 1920 × 1080 pixels and a refresh rate of 60 Hz. Head positions were maintained at 65 cm from the displaying monitor with a jaw and forehead support to minimize the head motion (Figure 1). Each participant was allowed to adjust the head support to their comfortable level.

### 2.2. Movement Pattern Design

**Figure 2:**
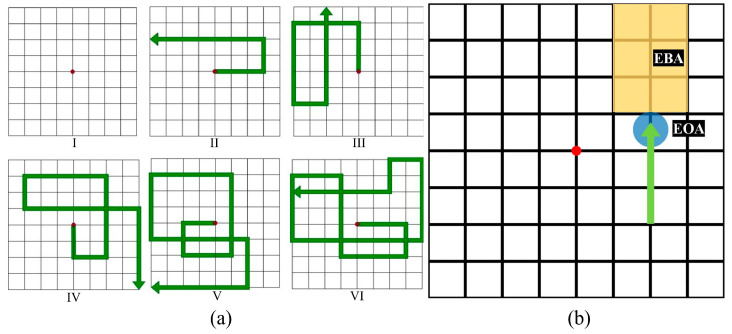
(**a**) Figure stimulus and the movement pattern of video stimuli. The moving arrowhead left a trail behind. When the arrowhead reached a new turning point, the previously shown trail vanished, and a new trail was generated through the arrowhead. (I) The figure stimulus was presented right before and after each video play, representing the origin by a red dot. (II), (III), (IV), (V), and (VI) represent the arrow movement trajectory of the video stimuli in Blocks 1, 2, 3, 4, and 5, respectively. (**b**) Illustration of the areas of interest (AOIs). Eye before arrow (EBA) was defined to compute early eye disengagement from the target. EBA is the yellow rectangular AOI positioned before the arrow tip. One of the AOI’s borders is located on the arrow’s tip. The AOI size of EBA was reduced as the arrow advanced and was deactivated when the arrow reached the next turning point. Eye on arrow (EOA) refers to the blue circular AOI consistently aligned with the arrow tip.

As shown in Figure 2a, the video stimuli were purely visual and displayed continuously with moving arrows on an 8-by-8 grid, with a red dot in the center indicating the arrow’s origin. When the video began, the arrows originated from the red central point of the grid and moved along the grid’s lines. The arrow traveled a certain distance and changed its direction at the grid intersections, which were referred to as turning points. The turning points in each video were unknown to the participant. We designed a cohort of videos with 3, 5, 7, 9, and 11 turning points, respectively (Figure 2a). The total length of the movement pattern in each video equals the number of turning points multiplied by 4 cell lengths. All arrows in the videos moved at a consistent speed of 1 cell length per second. Thus, the total duration of each video, in seconds, is determined by multiplying the number of turning points by 4 s and then dividing by the speed of 1 cell length per second. For each participant, the total time spent purely on the movement pattern was 280 s. The grid layout of the figure stimuli was identical to that of the video stimuli, with additional instructions presented on the right side of the screen.

### 2.3. Tracking Task Procedure

**Figure 3:**
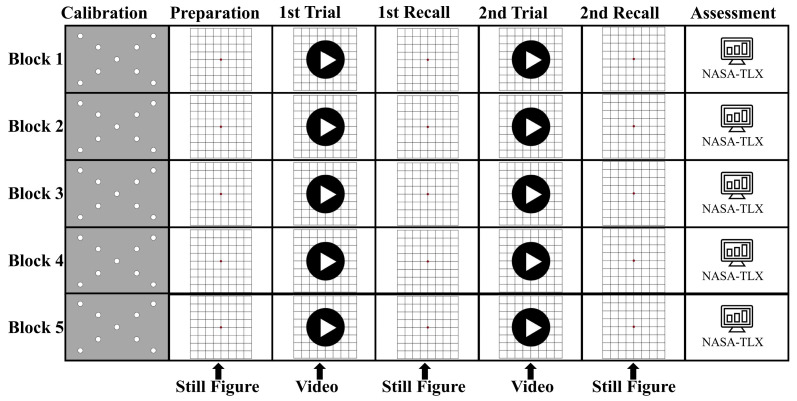
Task procedure from Block 1 to Block 5. Each block began with an eye-tracking calibration section, followed by the presentation of a still grid image. The still grid image was followed immediately by video stimuli when the participant was asked to track arrow movement for the first time. Following the video stimuli were recall stages. This stage involved the presentation of static grid image stimuli, during which the participants were specifically asked to recall the movement patterns they observed in the previous video.

When a participant entered the lab, we provided the participant with an orientation on how to perform the visual tracking task, followed by an introduction to the NASA-Task Load Index (NASA-TLX) [14]. After the orientation, visual tracking tasks were officially started. The visual tracking task was performed by playing videos of the five movement patterns. Participants were required to commit each pattern to memory and pay attention to the direction and distance because they were required to recall the movement pattern verbally immediately after watching each video. Figure 3 showed the experimental procedures. After eye-tracking calibration, participants were presented with a still grid image (Figure 2a(I)) that prepared them for the videos and directed their gaze to the center of the monitor. When the participant was ready, the experimenter terminated the still grid image and delivered the video stimulus for the arrow tracking task automatically.

The still grid image showed up again at the end of the video stimulus for participants to verbally recall the movement distance and direction they had just tracked. The video stimulus was displayed for the second time, followed by a verbal recall. Participants were then given a 1 min break before being asked to complete the NASA-TLX assessment. The above process refers to a block of tasks. We had a total of five blocks for each participant, showing them five arrow movement patterns, all of which were displayed in the Tobii Pro Lab (version 1.171.34991) for tracking participants’ eye movements. By having more turning points from Block 1 to 5, we introduced additional movement lengths and changes in direction, leading to a more complex task. Our aim was to challenge participants with a higher workload by making them monitor and recall more information. Participants were allowed to have sufficient rest time between blocks. The same steps were repeated until each of the five blocks was completed.

### 2.4. Measures

#### 2.4.1. Recall Accuracy Score (RAS)

In this experiment, the subjects’ responses during the verbal recall phase served as the basis for calculating the RAS. The experimenter compared participants’ answers to the correct movement at each turning point, which took the form of “distance—direction” (Example: Four Units-Down). One point was awarded if the participant’s answer matched the correct movement. No points were awarded if the recall did not match the correct movement, either by distance or direction. Because each block has a different number of turning points, the participant’s points were divided by the total number of turning points for each block before adding them together to produce a final RAS for subsequent data analysis. The calculation formula is Recall accuracy score = Points awarded/Total number of turning points. The recall accuracy was a value from 0 to 1, where 1 denotes the best recall accuracy.

#### 2.4.2. Eye-Tracking Metrics

Early eye disengagement was calculated from eye-tracking metrics by Tobii Pro Lab. We first defined two Areas of Interest (AOIs): one was Eye on arrow (EOA), and the other was eye before arrow (EBA) (shown in Figure 2b). These two AOIs were manually marked by the experimenter using the AOI function of Tobii Pro Lab. EOA was a circle (100 pixels in diameter) centered on the arrow’s tip that covered the arrow tip and was sufficient to capture fixations accurately. As the arrow in the video was constantly moving along the grid, the EOA was following the arrow tip (Figure 2b). The visual angle from the central point (center of the grid) to any of the four corners is approximately 14.9 degrees, with one cell length being roughly 2.6 degrees and the diameter of the EOA being about 2.2 degrees. EBA was a rectangular area in front of the arrow with a width of two cell lengths perpendicular to the arrow and a length from the tips of the arrow to the grid’s edge. The arrow was constantly moving to the turning point. The length of the rectangular area decreased as the arrow advanced (Figure 2b). The EOA border remained connected to the EBA border. According to Tobii pro nano specifications, the lag on calculating the AOI is only 1 frame (1/60 s).

Once we set up the above two AOIs, we extracted and examined the total number of fixations (times/s) and total duration of fixation (ms/s) within these two AOIs. Fixations are periods during which the eyes remain relatively still. This was calculated from a sequence of raw gaze points, where the estimated gaze velocity is below the velocity threshold (30 degrees per second) set in the Velocity-Threshold Identification gaze filter of Tobii.

EEDT was computed as the fixation metrics contained in EBA. Since the total duration of each block differed, the total number of fixations and total duration of fixation on each AOI (EBA or EOA) were averaged with respect to the task time to generate the mean frequency of fixation (FixFreq) and mean duration of fixation (FixDura), respectively, for the subsequent statistical analysis.

#### 2.4.3. Pre-Processing of Eye-Tracking Data

The pre-processing was finished in Excel by researcher Yun Wu under the supervision and review of Bin Zheng. We employed Tobii Pro Lab for the initial analysis and data exportation. The exported data included our fixation metrics of interest—the number and duration within each AOI—and was annotated with specific identifiers such as participant number, block number, and trial number. EOA is a continuous AOI, the metrics of which can be analyzed directly. The pre-processing primarily focused on organizing the fixation metrics from the multiple AOIs involving EBA. These measures were subsequently aggregated to calculate the EBA fixation metrics for each video play pertaining to each participant. The frequency was then defined as the total count of fixations divided by the overall task duration. We ultimately yielded the fixation frequency and duration within the EBA and EOA for each task session and participant for further analysis.

#### 2.4.4. NASA-TLX

NASA-TLX was utilized for assessing participants’ cognitive load after each block. NASA-TLX assessed cognitive load on these six domains: Mental Demands, Physical Demands, Temporal Demands, Effort, Frustration, and Performance [14]. A custom-designed software was used for participants to score each of the six domains and then answer a series of paired-choice questions. Each question presented two domains and asked participants to indicate which domain they considered more important. This allowed for the determination of the weight of each domain. The final NASA-TLX score was calculated by multiplying the value of each domain by its corresponding weight. The resulting score reflected both the individual domain scores and their perceived relative importance, providing an overall NASA-TLX score for further analysis.

### 2.5. Data Analysis

The statistical analysis was performed using R (version 4.2.1) and R studio. The EBA-FixDura and EBA-FixFreq values were transformed to achieve a normal distribution by taking the square root. A 5 × 2 (block: task complexity × trial) within-subject ANOVA was used for the comparison of the eye-tracking variables among five blocks and between two repeated trials. The Greenhouse–Geisser method was employed to adjust the lack of sphericity. We used the Pearson correlation coefficient to characterize the relations between our studied variables and visualized their relationships by displaying the scatter plots using the GGally package (Barret Schloerke et al., USA). The means and standard deviations were reported unless otherwise stated. A *p*-value < 0.05 was considered statistically significant.

## 3. Results

### 3.1. Participants

A total of 15 participants voluntarily participated in the study, with a mean age of 28 ± 7 years. The sample included 5 males and 10 females. Each participant completed the five blocks of tracking tasks, with each task being repeated twice. A power analysis of two-way within-subject ANOVA was performed in R for our primary outcome, EBA-FixDura. Using a subset of this sample, we obtained the eta (η) value for a block of 0.11, and the η value for a trial is 0.07. The current sample had a total of 15 participants, an equal sample size of 75 for a block and 30 for a trial. With an α of 0.05, the current sample had an effect size of 0.274 for a block and 0.352 for a trial, giving a power of 0.996. Our current sample size satisfies the requirement for sufficient power.

### 3.2. RAS

A two-way within-subject ANOVA was conducted on RAS and revealed the significant main effects on block (F _(4, 56)_ = 18.93, *p* < 0.001), trial (F _(1, 14)_ = 62.35, *p* < 0.001), and interaction (F _(4, 56)_ = 14.74, *p* < 0.001). Specifically, the RAS was significantly different among five blocks of task complexity (Block 1 = 1.00 ± 0.00, Block 2 = 0.97 ± 0.08, Block 3 = 0.71 ± 0.24, Block 4 = 0.69 ± 0.28, Block 5 = 0.63 ± 0.33). RAS in the second trial (0.90 ± 0.19) was significantly higher than in the first trial (0.71 ± 0.30). The main effect post hoc (Bonferroni) test shows differences between Block 1 and Blocks 2 to 5 and Block 2 and Blocks 3 to 5.

### 3.3. NASA-TLX Score

**Figure 4:**
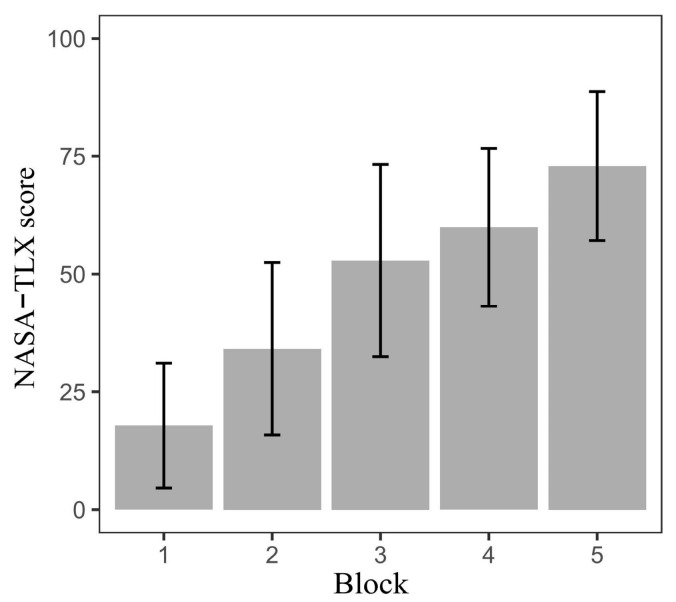
The NASA-TLX score increased from Block 1 to Block 5. The difference between blocks is statistically significant except between Blocks 3 and 4.

A one-way within-subject ANOVA was conducted on the NASA-TLX score and revealed significant main effects (t = 64.82, *p* < 0.001). NASA-TLX was significantly different between Block 1 (17.82 ± 13.04), Block 2 (34.16 ± 17.98), Block 3 (52.87 ± 20.04), Block 4 (59.91 ± 16.44), and Block 5 (72.91 ± 15.54). A post hoc (Bonferroni) analysis revealed statistical significance (*p* < 0.05) between every pair of blocks except for Blocks 3 and 4 (F= −1.86, *p* = 0.08). Figure 4 illustrates that the overall cognitive load rose from the first to the fifth block despite each respondent’s baseline level of the cognitive load differing.

### 3.4. Eye on Arrow

#### 3.4.1. EOA-FixDura

**Figure 5:**
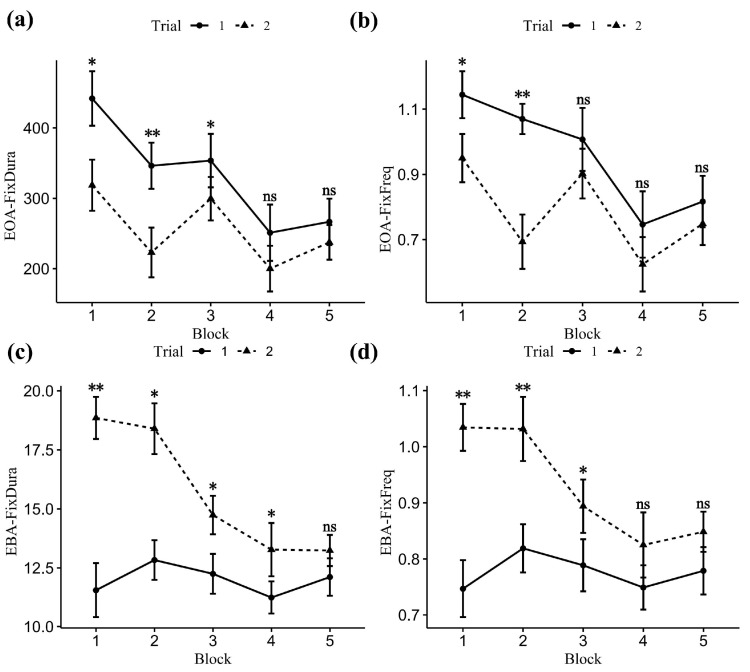
Line graph of the subjects’ eye-tracking metrics between blocks and trials. The medium of the error bar reflected the mean value of a group. The lower and upper limits of the error bar are defined as the mean ± standard error of the value from each group. Circular dots with solid lines indicate the first trial, and triangular dots with dashed lines indicate the second trial. (**a**) EOA-FixDura; (**b**) EOA-FixFreq; (**c**) EBA-FixDura; (**d**) EBA-FixFreq; ns, not significant, * *p* ≤ 0.05, ** *p* ≤ 0.001.

Within-subject ANOVA on EOA-FixDura revealed a significant main effect on block (F _(4, 56)_ = 10.87, *p* < 0.001), trial (F _(1, 14)_ = 31.37, *p* < 0.001), and interaction (F _(4, 56)_ = 2.90, *p* = 0.03). As the tracking task complexity increased, participants performed shorter EOA-FixDura (Block 1 = 380.25 ± 156.00 ms, Block 2 = 284.70 ± 144.08 ms, Block 3 = 326.56 ± 134.49 ms, Block 4 = 225.62 ± 141.15 ms, Block 5 = 252.09 ± 111.68 ms. The post hoc (Bonferroni) test showed significant differences between Block 1 and Block 2 (t = 3.77, *p* = 0.007), Block 4 (t = 5.74, *p* < 0.001), Block 5 (t = 4.78, *p* < 0.001); and between Block 3 and Block 4 (t = 6.29, *p* < 0.001), Block 5 (t = 4.05, *p* = 0.003). Repeating the task shortened the EOA-FixDura (Trial 1 = 331.92 ± 154.26 ms, Trial 2 = 255.76 ± 129.65 ms). The interaction between Block and Trial is shown in Figure 5a. Specifically, repeating the trial helped to shorten the EOA-FixDura; this effect was more dramatically observed in Block 1, Block 2, and Block 3 when task complexity was low.

#### 3.4.2. EOA-FixFreq

Within-subject ANOVA on EOA-FixFreq revealed a significant main effect on block (F _(4, 56)_ = 8.04, *p* < 0.001), trial (F _(1, 14)_ = 45.87, *p* < 0.001), and interaction (F _(4, 56)_ = 3.66, *p* = 0.02). As the tracking task complexity increased, participants performed a smaller number of EOA-FixFreq (Block 1 = 1.05 ± 0.30, Block 2 = 0.88 ± 0.32, Block 3 = 0.96 ± 0.34, Block 4 = 0.69 ± 0.36, Block 5 = 0.78 ± 0.28. The post hoc (Bonferroni) test showed significant differences between Block 1 and Block 4 (t = 5.66, *p* < 0.001), Block 5 (t = 4.19, *p* = 0.002); and between Block 3 and Block 4 (t = 5.42, *p* < 0.001), Block 5 (t = 3.37, *p* = 0.021). Repeating the task decreased the EOA-FixFreq (Trial 1 = 0.96 ± 0.34, Trial 2 = 0.78 ± 0.32). The interaction between block and trial is shown in Figure 5b. Specifically, repeating the trial helped to reduce EOA-FixFreq; this effect was more dramatically observed in Block 1 and Block 2 when task complexity was relatively low.

### 3.5. Eye before Arrow

#### 3.5.1. EBA-FixDura

Within-subject ANOVA on EBA-FixDura revealed a significant main effect on block (F _(4, 56)_ = 9.74, *p* < 0.001), trial (F _(1, 14)_ = 144.19, *p* < 0.001), and interaction (F _(4, 56)_ = 8.43, *p* < 0.001). As the tracking task complexity increased, participants performed a shorter fixation before the arrow: EBA-FixDura also differed significantly between Blocks 1 (15.20 ± 5.39), 2 (15.61 ± 4.64), 3 (13.49 ± 3.40), 4 (12.26 ± 3.70), and 5 (12.67 ± 2.83). The post hoc (Bonferroni) test showed significant differences between Block 2 and Block 3 (t = 3.12, *p* = 0.04), Block 4 (t = 3.91, *p* = 0.005), Block 5 (t = 3.28, *p* = 0.027). Repeating the task prolonged the EBA-FixDura (Trial 1 = 12.00 ± 3.35, Trial 2 = 15.70 ± 4.28). The interaction between block and trial is shown in Figure 5c. Specifically, repeating the trial helped to increase EBA-FixDura; this effect was more dramatically observed in Block 1 and Block 2 when task complexity was relatively low.

#### 3.5.2. EBA-FixFreq

Within-subject ANOVA on EBA-FixFreq revealed a significant main effect on block (F _(4, 56)_ = 6.90, *p* < 0.001), trial (F _(1, 14)_ = 91.57, *p* < 0.001), and interaction (F _(4, 56)_ = 4.52, *p* = 0.003). As the tracking task complexity increased, participants performed a smaller EBA-FixFreq (Blocks 1 (0.89 ± 0.23), 2 (0.93 ± 0.22), 3 (0.84 ± 0.19), 4 (0.79 ± 0.19), and 5 (0.81 ± 0.15). The post hoc (Bonferroni) test showed significant differences between Block 2 and Block 4 (t = 3.32, *p* = 0.002). Repeating the task made the participant perform a larger EBA-FixFreq (Trial 1 = 0.78 ± 0.17, Trial 2 = 0.93 ± 0.21). The interaction between block and trial is shown in Figure 5d. Specifically, repeating the trial helped to increase EBA-FixFreq; this effect was more dramatically observed in Block 1, Block 2, and Block 3 when task complexity was relatively low.

### 3.6. Pearson Correlation Analysis

**Figure 6:**
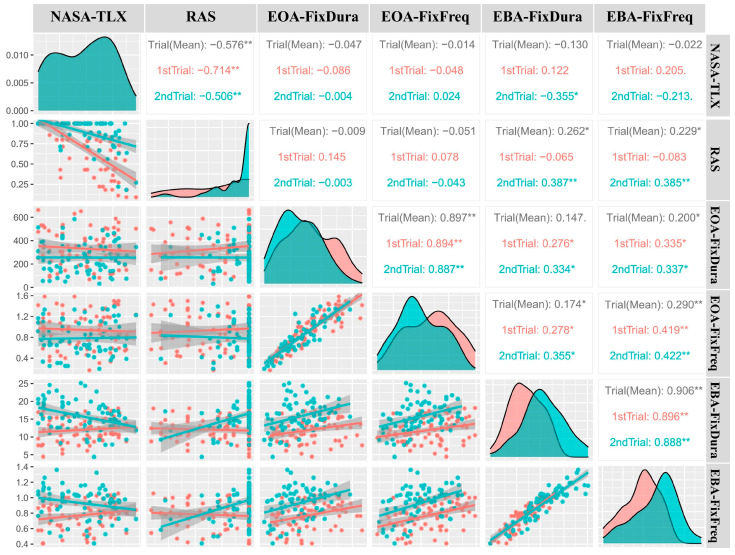
The Pearson correlation coefficient and scatter plot between variables. The scatter plots and best-fit lines between two variables are displayed in the lower left section. The shaded areas represent the 95% confidence intervals of the regression line. The corresponding r values for the two-trial average, as well as for the first trial (red) and second trial (green), are displayed in the upper right section of the figure. The data distribution for that variable is displayed in the diagonal zone. All variables, except for recall accuracy score (RAS), are approximately normally distributed. *: *p* < 0.05 and **: *p* < 0.001.

Pearson correlations were performed among the variables as we intended to investigate the association between eye behaviors, recall accuracy, and perceived workloads (Figure 6). We also created scatter plots to visualize the relationship between them (Figure 6). Within each AOI (EOA or EBA), FixFreq and FixDura were significantly and strongly correlated with one another (EOA: r = 0.89, *p* ˂ 0.001; EBA: r = 0.91, *p* ˂ 0.001)). There was also a negative correlation between the NASA-TLX score and the RAS (r = −0.58, *p* ˂ 0.001) for both trials. EOA-FixDura and EOA-FixFreq had no statistically significant correlations with NASA-TLX (FixDura r = −0.05, FixFreq r = −0.01) or recall accuracy (FixDura r = −0.00, FixFreq r = −0.05). EOA-FixDura had a borderline significant correlation with EBA-FixDura (r = 0.15, *p* = 0.07) and a significant correlation with EBA-FixFreq (r = 0.20, *p* = 0.01). EOA-FixFreq is significantly correlated with EBA-FixDura (r = 0.17, *p* = 0.03) and EBA-FixFreq (r = 0.29, *p* ˂ 0.001). The EBA-FixDura had a significant positive correlation with the RAS (r = 0.39, *p* ˂ 0.001) and a negative correlation with the NASA-TLX score (r = −0.36, *p* = 0.002) for the second trial (all with medium strength). In addition, EBA-FixFreq was significantly and moderately correlated with the RAS (r = 0.39, *p* ˂ 0.001).

## 4. Discussion

Investigating EEDT provides a deeper understanding of how individuals adjust/react to environmental uncertainty. In this study, we sought to explore how task complexity and task repetition influence EEDT in a pure experimental environment. Our findings have validated our three hypotheses, showing a notable effect of task complexity on reducing EEDT and repetition on increasing it. Interestingly, the one-time repetition’s influence on EEDT was significant only at lower task complexities and became negligible as the task complexity increased.

When tracking moving objects, prior research has identified several associated parameters, including pursuit gain, target velocity, latency, and acceleration [15,16]. Such parameters critically assess one’s capability of tracing moving objects seamlessly. In our study, we have used fixation-related parameters to describe the EEDT because fixation of eye tracking meaningfully represents a human’s visual attention. Also, they can be accurately quantified and compared across groups. Fixation-related parameters have also been extensively used in tasks that involve continuous movement to describe EEDT and its synonyms. For instance, when making a basketball shot, the eye predominantly fixes on the target, more so than the ball’s trajectory, with engagements typically identified through fixations exceeding 100 ms around the hoop [17,18]. Similarly, in dart throws, fixation duration on the target serves as a primary metric, correlating with the throw’s precision [19]. The trend continues in babies, where the first fixation on a goal-directed AOI is the onset of anticipatory eye behavior [20]. Fixation allowed us to quantify such EEDT, especially by examining the variations under different task conditions.

We managed task complexity in the visual tracking task by regulating object moving steps and directions. To make sure this setting would alter participants’ perception of task loads, we asked participants to perform NASA-TLX evaluations at the end of every block. The NASA-TLX score was found to be increasing significantly as the visual tracking task involved more steps and direction changes. We found that as task complexity increased, the recall accuracy of EEDT decreased, measured by a smaller fixation frequency and shorter fixation duration in the EBA. The result of this study aligned with the previous reports, where individuals with spare cognitive resources were capable of processing information from the upcoming target [21,22]. The EEDT decreased as the current task complexity/cognitive load increased because fewer spare cognitive resources (maybe in working memory) were available. In driving tasks, drivers performed less anticipatory eye scanning when a higher number of loads were placed in their working memory. Such visual anticipation could be interpreted as a competition for working memory resources [22]. A linguistic study also revealed that reduced working memory capacity limited anticipatory eye gaze during sentence processing, which was also consistent with our results [21].

The impact of repetition on the performance of EEDT was opposite to the task complexity. As participants were gaining repetition on the task, they performed more EEDT. Working memory capacity theory provides a probable explanation for this observation [21,22]. Prior learning generated from repetition decreases the amount of information needed to be processed and frees up more spare cognitive resources in the working memory for performing EEDT. The repeated trials might also reduce the uncertainty about moving trajectories, which would facilitate the anticipation of the upcoming path before the arrow (target). Moreover, these results are supported by previous findings on EEDT in surgical settings, in which experienced surgeons performed more EEDT [8,13].

Interestingly, we observed an antagonistic interaction between task complexity and task repetition. Because the repetition was a single time, the effect of task repetition on anticipatory eye movements was limited by task complexity. The beneficial effect of task repetition seemed to work only when task complexity was relatively low (i.e., Blocks 1 and 2, and to some extent for Blocks 3 and 4). The reduced beneficial effects of task repetition when task complexity is high may also be explained by theory: high-complexity tasks would occupy a larger amount of working memory than can be released by task repetition, thus depleting the working memory capacity released by task repetition to increase EEDT [23,24]. For more complex blocks, more repetition may be needed to achieve the effects.

Typically, EEDT is a measure of eye–hand coordination and is linked to skill expertise [2,4,25,26]. Eye disengagement from the current task and early engagement (fixation) to a future one serves as a guiding function by acquiring information about the target and environment in advance and subsequently passing it back to the brain’s motor control system, which then accurately and effectively guides body movements [1,7]. On the one hand, when individuals improved at goal-directed movement, they would get better at disengaging their eyes to optimize gazing in accordance with the precise time and location where a future event is anticipated to occur [27,28]. On the other hand, the intentional training of eye engagement has emerged as an effective method for improving both gaze focus and motor performance [29]. This study is distinct from prior research [8] in that it examines how eye disengagement is regulated by multiple levels of incremental complexity in a rigorously controlled experiment using a novel approach to define EEDT. Our study supported and enhanced previous understanding by revealing the EEDT pattern associated with task complexity and repetition. However, such a pattern was observed in visual tracking tasks; we are uncertain whether a similar pattern remains in actual goal-directed movements. We will continue to investigate this phenomenon in a task involving hand movement.

In the correlation analysis (Figure 6), a significant negative association existed between NASA-TLX and RAS, confirming previous observations between workloads and task recall accuracy (Figure 6) [30]. We have seen a strong coupling between fixation frequency (FixFreq) and fixation duration (FixDura) when participants performed EEDT. Importantly, we discovered that EEDT was moderately (r > 0.3) correlated to RAS and the NASA-TLX score. These findings suggest that eye-tracking metrics, especially EEDT, are valuable for assessing task-induced cognitive load and recall accuracy. The trends depicted in Figure 6 are logically consistent with those in Figure 5. Specifically, in Trial 2, EBA-FixDura exhibits a significant (*p* < 0.05) negative linear relationship with NASA-TLX, which is consistent with the trend observed in Figure 5c. There is no significant correlation between EBA-FixDura and NASA-TLX in Trial 1, which is also consistent with the trend observed in Figure 5c. Similarly, in Figure 6, EBA-FixFreq exhibits a visible negative trend in Trial 2 and a slight positive trend in Trial 1, although without statistical significance, which is consistent with the general trend in Figure 5d. As indicated by the trends shown in Figure 5a,b, the correlation between NASA-TLX and EOA-related metrics may be better modeled by other regression models instead of simple linear ones. This could be the reason for the lack of significance.

Monitoring in-task cognitive load is important for avoiding negative consequences and improving skill acquisition in high-risk situations. Numerous studies have reported that eye behaviors could be a timely and cost-effective tool for evaluating human cognitive load. For instance, the magnitude of the microsaccade and the diameter of the pupil were both reliable indicators for measuring cognitive load [31]. Cognitive load may also be reflected in the fixation duration. In different task settings, it was reported that as cognitive load increased, fixation duration either increased [32,33] or decreased [34,35,36]. Evidence from this study suggests that EEDT could potentially be used in the future to objectively evaluate cognitive load.

Eye-tracking devices represent an important sensor technology for the future analysis of EEDT in a variety of occupational settings. The EEDT-related findings can serve as promising sensor-based evidence with significant application in human factor assessment and cognitive support for trainees [37]. The selected arrow movement pattern for this task has a theoretical basis in the literature. Arrows are a common stimulus in daily life, particularly in activities that require spatial navigation. Dalmaso et al. conducted a study that demonstrated how, despite being non-social cues, arrows exert a similar influence on eye movements as biological or social stimuli. They are processed with a similar spatial attentional priority. The results of arrow-tracking tasks can provide insights into the mechanisms of attentional control in real-world scenarios. Consequently, this study has practical applications in areas ranging from navigation systems to digital interface design [38].

This study had some limitations. The first was the definition of the AOI. This study defined two AOIs, EOA and EBA, which did not exhaust the analysis of more diverse AOI configurations in the task interface. There may be fixations on other areas, such as the areas on the left and right, which may reveal important behavioral correlations. This represents a promising avenue for future research. Secondly, the use of a randomized task order may have been more effective than the current measures of randomizing the movement pattern and arranging break time between blocks in reducing potential confounding factors such as practice and fatigue. Thirdly, subjects were only asked to perform a single repetition. The effects of repeating the task multiple times could not be observed. For a more complete understanding of learning effects, more repetitions would be desirable in the future. Finally, although we used a well-established eye-tracking device, the accuracy and limitations of the eye tracker may introduce some variance in the data analysis.

## 5. Conclusions

EEDT is a critical and common eye behavior for planning and coordinating actions. In this study, we found direct evidence of how EEDT changes with an increase in task complexity and task repetition in a visual tracking task. Task complexity decreased individuals’ EEDT performance, whereas task repetition increased it. Interaction between these two independent variables suggests the beneficial effect of one repetition seems to have worked when task complexity was relatively low. This study also expanded our understanding of how eye behaviors correlate with task performance and cognitive loads. It provides meaningful sensory evidence for developing more advanced eye-tracking sensors and human factor assessment.

## Data Availability

The datasets analyzed in the current study are available from the corresponding author upon reasonable request.
